# A Fatigue Crack Size Evaluation Method Based on Lamb Wave Simulation and Limited Experimental Data

**DOI:** 10.3390/s17092097

**Published:** 2017-09-13

**Authors:** Jingjing He, Yunmeng Ran, Bin Liu, Jinsong Yang, Xuefei Guan

**Affiliations:** 1 School of Reliability and Systems Engineering, Beihang University, Beijing 100191, China; hejingjing@buaa.edu.cn (J.H.); rym2015@buaa.edu.cn (Y.R.); 2 China Academy of Launch Vehicle Technology, Beijing 100071, China; yangjinsong@buaa.edu.cn; 3 Siemens Corporation, Corporate Technology, 755 College Rd. E., Princeton, NJ 08540, USA; xuefei.guan@siemens.com

**Keywords:** Lamb wave simulation, crack size quantification model, Bayesian updating, uncertainty

## Abstract

This paper presents a systematic and general method for Lamb wave-based crack size quantification using finite element simulations and Bayesian updating. The method consists of construction of a baseline quantification model using finite element simulation data and Bayesian updating with limited Lamb wave data from target structure. The baseline model correlates two proposed damage sensitive features, namely the normalized amplitude and phase change, with the crack length through a response surface model. The two damage sensitive features are extracted from the first received S_0_ mode wave package. The model parameters of the baseline model are estimated using finite element simulation data. To account for uncertainties from numerical modeling, geometry, material and manufacturing between the baseline model and the target model, Bayesian method is employed to update the baseline model with a few measurements acquired from the actual target structure. A rigorous validation is made using in-situ fatigue testing and Lamb wave data from coupon specimens and realistic lap-joint components. The effectiveness and accuracy of the proposed method is demonstrated under different loading and damage conditions.

## 1. Introduction

Structural health monitoring (SHM) is an integrated approach combining data acquisition and interpretation. It provides a means for damage status assessment and remaining useful life prediction [1,2,3,4]. In recent years, guided ultrasonic waves have gained widespread applications in the field of SHM as a method of non-destructive evaluations (NDE) due to its ability of traveling large distances in structures with little energy loss [5,6,7,8,9]. Lamb wave is one of the popular guided waves and has been identified as an effective tool to detect damages in plate-like structures. Lamb wave is a form of elastic perturbation that can remain guided between two parallel free surfaces, such as the upper and lower surfaces of plates or shells. The fundamental idea behind Lamb wave based damage detection is that discontinuities such as cracks and delamination in the wave path will alter characteristics of the wave. Lamb wave propagation properties such as amplitude, velocity and phase shift depend on both test frequency and material thickness. Those properties are expected to change due to the existence of structural defects. Lamb wave-based NDE has been extensively investigated for damage identification in metallic and composite materials [10,11,12]. Souza presented a method based on Lamb wave and a circular array of sensors for assessing faults in aluminum plates [13]. A damage index was defined from the analysis of the first package of received Lamb wave signal. Michaels presented a damage index called time domain difference which scales the Lamb wave signal to unity energy in order to evaluate the amplitude difference between damaged signal and healthy signal [14]. Zima proposed a diagnostic system dedicated for plate structures with variable lengths of linear cracks using guided wave-based techniques and the ellipse-based binary damage imaging algorithm, which has a great potential for damage growth evaluations in structural monitoring systems [15].

Despite a number of studies on Lamb wave damage detection have been reported in existing studies, there are still some challenges for engineering applications [16,17]. First, a great number of experimental datasets of Lamb wave testing are required to establish a reliable and accurate crack size quantification model. This is due to the fact that Lamb wave propagation properties are sensitive to local geometry features of the target structure, and there is no universal model available for direct applications in an given arbitrary target structure. However, such experiments may not be practical due to time and economic constrains. Meanwhile, Lamb wave data collected in real engineering structures are often contaminated by operational and environmental noises, which further increase the difficulty of achieving accurate damage quantification results. Therefore, instead of acquiring test data from the target structures for model development, a few studies used finite element (FE) simulation to investigate the underlying mechanisms for Lamb wave damage detection in order to minimize the required experimental work [18,19]. Hong proposed an approach to locate fatigue damage using nonlinear Lamb wave. In the method, FE simulations were used to obtain the linear and nonlinear damage indices from the Lamb wave testing data [20]. Vanli proposed a probabilistic method using both FE simulations and sensor data to predict the extent and location of damages [21]. In the study, the damage index calculated from finite element model was utilized as a bias correction function for damage size. De Fenza reported the propagation of Lamb wave in metallic and composite plates using FE simulations [22]. A damage index was defined as a determinant of structural damage by magnitudes of spectra for the damaged and undamaged structures. The damage index was shown to have a direct relationship with the crack size in the plates. Shen and Giurgiutiu investigated the FE model of Lamb waves interacting with linear notch cracks and nonlinear breathing cracks. The result of FE simulation agrees well with the analytical model [23]. Agrahari and Kapuria studied a damage detection strategy based on the time-reversal process (TRP) of Lamb waves [24]. The time reversibility of Lamb waves in metallic plates were studied using both experiments and FE simulations. The reported studies have shown that FE simulation is an effective tool to simulate Lamb wave responses of damages to support the development of damage detection and assessment methods. However, most of the existing studies using FE simulations focus on specific targets and the resulting methods cannot directly be applied to other structures. On the other hand, differences between FE simulation and field testing, such as boundary conditions, material properties, loading conditions and other factors, may have a significant influence on the accuracy of the damage quantification model. Therefore, obtaining a general and reliable damage quantification model using FE simulations is still a practical challenge.

This study has two objectives. The first one is to establish a general methodology for a reliable, effective and accurate Lamb wave-based crack size quantification model using FE simulation data and Bayesian updating. The focus is to investigate whether using FE simulation data from a numerical model can be used to replace the process of acquiring training data from actual components. The uncertainty of interest is between the numerical model and the realistic structures. It is different from the previous study reported in Ref. [10] where the uncertainty of interest is the inter-component uncertainty of physical components with different dimensions and geometry features. The motivation is to further reduce the economic impact on acquiring necessary data for building the baseline crack size quantification model. The second is to investigate the influence of uncertainties to the proposed method for realistic applications. The rest of the study is structured as follows. First, the procedure of finite element model simulation of Lamb wave propagation in an aluminum plate is presented. Two sensitive features, namely the normalized amplitude and the phase change, are proposed based on the physical mechanisms of Lamb wave interactions with cracks. Next, a baseline crack size quantification model is established using the FE simulation data. The crack size is correlated with the two damage sensitive features through a proposed response surface model. To account for the difference between numerical experiments and Lamb wave data from actual target structures, Bayesian updating is proposed to calibrate the initial baseline model with sparse measurement data from the target structures. Following that, a rigorous validation of the proposed method is made using a large set of experimental data acquired from coupon specimens and realistic lap-joint components in an in-situ fatigue testing environment. Uncertainties from numerical modeling, loading, material, and manufacturing are systematically considered in the validation process. The effectiveness and accuracy of the proposed method is demonstrated. Finally, conclusions are drawn based on the current study.

## 2. Finite Element Modeling of Lamb Wave Propagation

**Figure 1:**
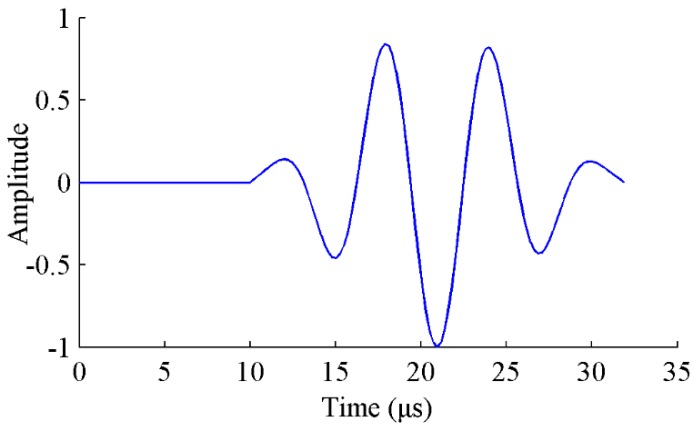
The 3.5-cycle excitation signal for Lamb wave.

The simulation of Lamb wave propagation is a highly computational demanding task. It requires both a fine temporal and spatial discretization to capture the different wave modes. To alleviate the computational hurdle, semi-analytical finite element methods (SAFE) [25] and wave finite element methods (WFE) [26,27] were developed to offer fast and accurate results. However, these methods are difficult to analyze complex three-dimensional geometries and arbitrary perturbations of the waveguide. The wave propagation in structures containing discontinues, such as delamination, cracks or other defects, are hard to be described analytically [28]. There are mainly two ways to simulate Lamb wave propagation in plate-like structures using FE models. In the first method, both the piezoelectric (PZT) transducers and the host structures are modeled [23,24,29]. The PZT transducers are modeled with piezoelectric elements. A perfect bonding between the plate and PZT is assumed. A voltage input of tone burst signal is applied to the top surface of the PZT sensor as an excitation [24]. For signal sensing, the equipotential condition of the electrode surface of the sensor is imposed by coupling the electric potential degrees of freedom (DOFs) of the surface. For the second method, the input signal is equivalently represented by applying vertical forces or uniform in-plane radial concentrated forces on the nodes of sensors [30,31]. The tone burst can also be excited as the out-of-plane displacement [32]. The coupling effect between the PZT transducer and the host structure is therefore no needed in this method. Both methods have been proven to be consistent with experimental results based on reported studies. Finite element models including PZT transducers can be computational expensive and time consuming. Therefore, the vertical displacement of sensor central point is employed as the input signal in this study. As shown in Figure 1, a 3.5-cycle tone burst is used in this study as an excitation signal. Additionally, a Hamming window function is adopted to concentrate the maximum amount of energy at the desired driving frequency.

**Figure 2:**
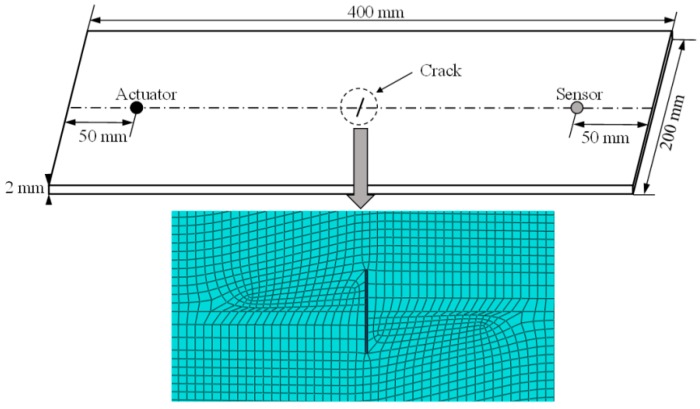
The dimension of the plate structure, crack location, and mesh of the 3D finite element model.

The dimension of the modeled aluminum plates is 400 mm × 200 mm × 2 mm, as shown in Figure 2. The propagation of Lamb wave is known to be dispersive in nature. The velocity of Lamb wave propagation depends on not only the elastic constants and density of the material, but also the frequency of waves. Theoretically, multiple Lamb wave modes can exist simultaneously in plate-like structures, and the number of modes increases with the frequency. It is known that S_0_ mode of Lamb wave has a significantly larger wave length than the thickness of the plate; therefore, it has advantages over other modes regarding the sensitivity to smaller damages [33]. In addition, the group velocity of S_0_ mode wave is faster than that of the A_0_ mode wave. The first received S_0_ wave package has the least interrupted signal from boundary reflection and is more distinguishable. In this paper, the first wave package of S_0_ mode in the received signal data is employed to extract the damage sensitive features. The excitation frequency of the Lamb wave is set to be 0.16 MHz given the product of the thickness and excitation frequency is 0.32 MHz mm. The group velocity of S_0_ mode remains constant with a slight dispersion. 

The mechanical properties of the plate are listed in Table 1. A through-thickness crack with a 0.3 mm width is modeled in the center of plate. Crack sizes varying from 1 mm to 30 mm with an increment of 1 mm are used. A plate with no crack is also modeled as a reference. An actuator and a receiver with a distance of 300 mm are assumed on each side of the crack. The plate model is meshed with C3D8R solid elements as displayed in Figure 2. The four lateral sides of the plate model are fixed and the non-reflective boundary condition is used.

As Lamb waves travel in a plate, the longitudinal waves travel in the direction of wave propagation and transverse waves oscillate perpendicular to the direction of wave propagation. The wave propagation depends on the properties of medium, and the transverse wave velocity and longitudinal wave velocity of isotropic material are expressed as

(1) $$c_{T}=\sqrt{\frac{E}{2\rho (1+\upsilon )}},$$

and

(2) $$c_{L}=\sqrt{\frac{E(1-\upsilon )}{\rho (1-2\upsilon )(1+\upsilon )}},$$

respectively. The term *E* is Young’s modulus, *ν* is Poisson’s ratio, and *ρ* is density. The transverse wave velocity and longitudinal wave velocity given by Equations (1) and (2) are 3120.3 m/s and 6194.6 m/s, respectively. The smallest wavelength (*λ*_min_) is

(3) $$\lambda_{\min}=\frac{c_{T}}{f_{\max}}=\frac{3120.3\;m/s}{160\;\mathrm{kHz}}=19.5\;\mathrm{mm},$$

where *f* is the frequency of Lamb wave. The size of the elements is chosen in a manner so that the propagating waves can spatially be resolved. For this purpose, usually more than 10 nodes per wavelength are required. The typical element edge length can be determined as

(4) $$L_{e}<\frac{\lambda_{\min}}{10}=1.95\;\mathrm{mm}.$$

The minimum element edge length is chosen as 1 mm. The medial axis algorithm is used to mesh the area near the crack with regular-shaped mesh elements. The plate model without crack has 160,000 mesh elements and 241,803 nodes. The plate model with a crack has a variable number of elements and nodes depending on the crack size, for example, the plate with 5 mm crack has 157,656 mesh elements and 238,296 nodes.

For time integration, the accuracy of the model can generally be improved with an increasingly smaller integration time step. A large time step cannot resolve the high frequency components, and a small time step can lead to a significant computational demand. Therefore, a proper time step is required. In this paper, the time step (Δ*t*) selection follows two criteria in Ref. [34]. The CFL condition (Courant–Friedrichs–Lewy condition) ensures that there are at least 20 time steps during the cycle of a wave at the highest frequency to prevent a longitudinal wave from traveling completely through an element during a single time step. The time step calculation can be expressed as

(5) $$\Delta t<\frac{1}{20f_{\max}}=\frac{1}{20\times 160\;\mathrm{kHz}}=0.3125\;\mu s.$$

Another criterion given by Moser uses the minimum element edge length and longitudinal wave velocity to obtain the minimum time step [35]

(6) $$\Delta t<\frac{L_{\min}}{c_{L}}=\frac{1\;\mathrm{mm}}{6194.6\;m/s}=0.161\;\mu s.$$

**Figure 3:**
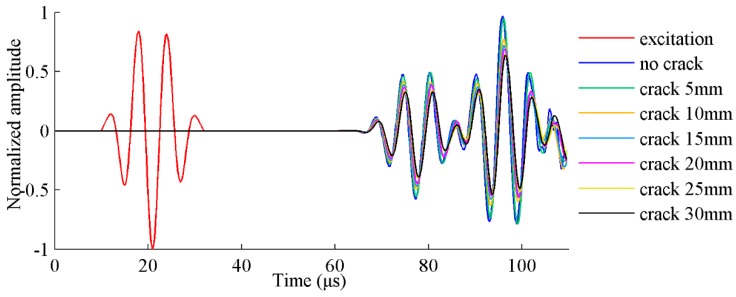
FE simulation of Lamb wave responses in a plate with different crack lengths.

The maximum time step is set to 0.1 μs considering Equations (5) and (6). FE simulation results of the excitation and receiving sensor locations are obtained and shown in Figure 3. The damaged plates are modeled with different crack sizes ranging from 5 mm to 30 mm. The data from the healthy plate without crack are also presented for comparison purposes.

## 3. Baseline Model of Lamb Wave Damage Detection Using FE Model

### 3.1. Signal Processing

**Figure 4:**
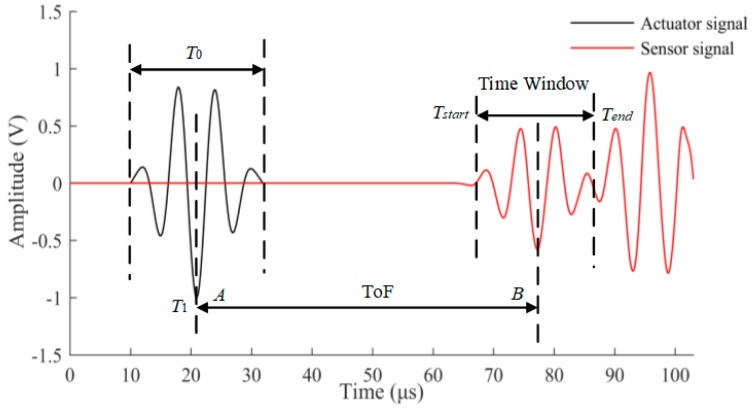
Illustration of the time window calculation of Lamb wave response data.

**Figure 5:**
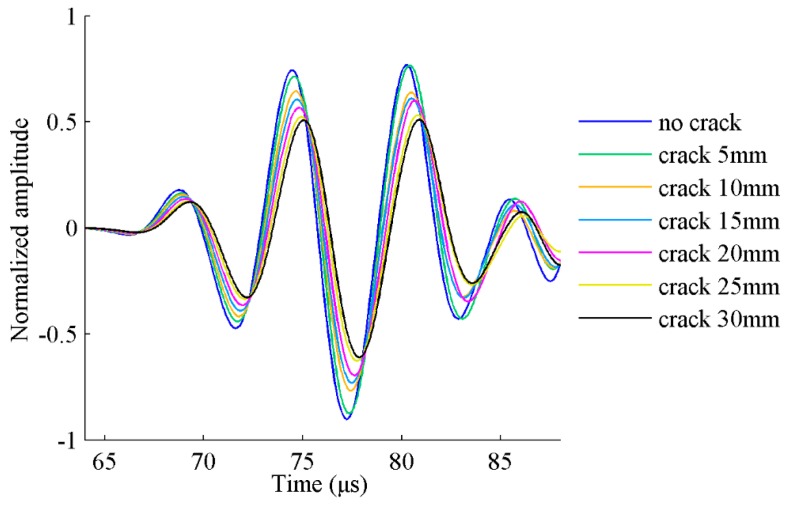
Lamb wave response data for healthy and damaged plates in the desired time window.

**Figure 6:**
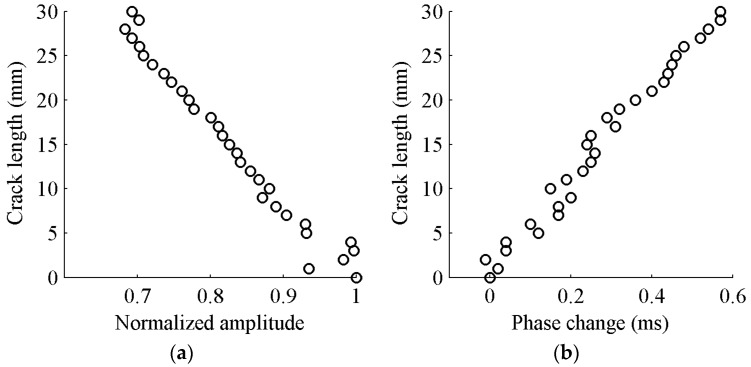
Crack length versus damage sensitive features: (**a**) normalized amplitude; and (**b**) phase change.

As mentioned earlier, the first S_0_ wave package is selected to determine the targeted time window for damage detection. The demonstration of time-of-flight (*ToF*) and the time window are shown Figure 4. The group velocity of Lamb wave is experimentally obtained as 5338 m/s. The time window of the first wave package is defined as the time duration between the start time point (*T_start_*) and the end time point (*T_end_*) of the response signal data. The response signal data in the selected time window are presented in Figure 5. It can be seen that the amplitude of received signal data gradually reduces as the crack size increases. This can be explained that a larger crack size can cause more energy loss when the wave propagates across it. The ratio of damaged signal amplitude to healthy signal amplitude is defined as normalized amplitude. For a notch or an open crack with no contact between two crack surfaces, the received signal contains the scattered waves in a detour route from the crack tip. The detour route results in a phase change of received signal; therefore, the phase change is considered as the time difference between the damaged plate and the healthy plate. The phase change of the received signal data becomes larger due to the fact that the traveling distance of the wave is longer for a longer crack. The crack length versus the normalized amplitude and phase change are shown in Figure 6a,b, respectively. A general monotonic relationship can be observed from the plots. Considering the above two physical mechanisms, the phase change and normalized amplitude are used as damage sensitive features to correlate the crack size in this study.

### 3.2. Baseline Crack Size Quantification Model

**Figure 7:**
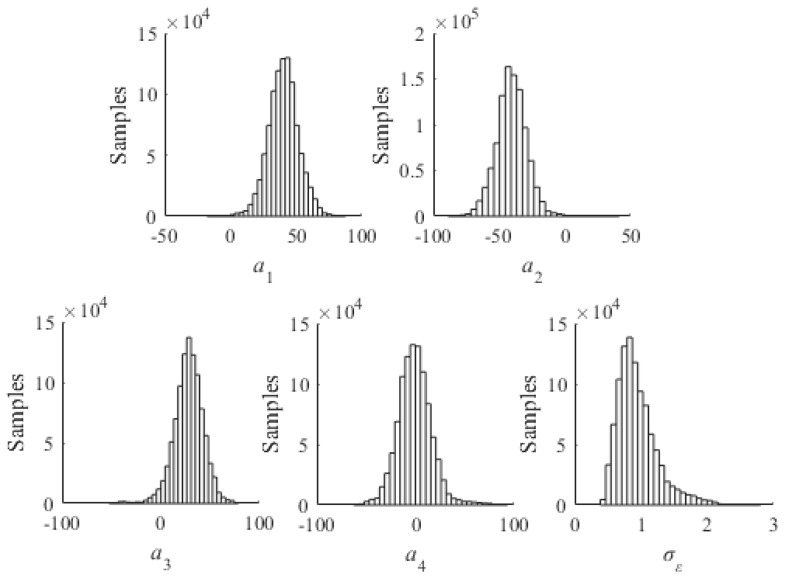
Histograms of prior model parameters estimated using FE simulation data.

To quantify the crack length, a regression model, with the damage sensitive features as model independent variables, needs to be developed. According to previous study, a single feature is difficult to quantify the damage accurately [36]. A response surface model incorporating the normalized amplitude, phase change, and the interaction between the two is established as

(7) $$a=a_{1}+a_{2}x+a_{3}y+a_{4}xy$$

where *a* is crack length, *a*_1*,…*,4_ are model parameters, *x* is the normalized amplitude, *y* is the phase change, and *xy* is the interaction between the two damage sensitive features. The method of Bayesian Estimator is used to identify parameter statistically. Consider the difference of model prediction for crack length and the actual measurement as a zero-mean Gaussian variable, *e*, with a standard deviation of *σ_ε_*, the joint distribution of the model parameter *θ* = (*a*_1_,*a*_2_,*a*_3_,*a*_4_) and *e* writes

(8) $$p_{0}\left(\theta ,\sigma_{\varepsilon }\right)\propto \frac{1}{\sigma_{\varepsilon }}{\left(\frac{1}{\sqrt{2\pi }\sigma_{\varepsilon }}\right)}^{n}\exp\left\{-\frac{1}{2}\sum_{i=1}^{n}{\left[\frac{a_{i}-a_{1}-a_{2}x_{i}-a_{3}y_{i}-a_{4}x_{i}y_{i}}{\sigma_{\varepsilon }}\right]}^{2}\right\},$$

where *a_i_* is the *i-*th measurement of crack length, *x_i_* and *y_i_* are the corresponding values of the normalized amplitude and the phase change, respectively, and *n* is the total number of data points. The leading term of Equation (8) is the non-informative prior of *σ_ε_* and the remaining term is the likelihood function. A total number of 1,000,000 samples are drawn from Equation (8) using Markov-Chain Monte-Carlo (MCMC) method with Metropolis-Hastings algorithm [37,38,39]. The resulting samples of the prior distributions of model parameters are presented as histograms in Figure 7. Basic statistics such as the mean vector and covariance matrix of (*a*_1_,*a*_2_,*a*_3_,*a*_4_) can trivially be calculated from the samples. A deterministic baseline model can be expressed using the mean vector of (*a*_1_,*a*_2_,*a*_3_,*a*_4_) as

(9) a=35.2813−34.6471x+41.6452y−14.5872xy.

It should be noted that the damage sensitive features (normalized amplitude and phase change) have specific physical meaning, and the baseline model obtained using FE simulation data reflects an ideal interaction of Lamb wave with cracks. It reflects the interaction from a numerical experiments point of view. In addition, the FE simulation is performed in a deterministic framework, which cannot account for uncertainties caused by the influencing factors in the real testing. Factors that affect the outcome of the Lamb wave damage detection can be classified into three groups, input parameters (geometry of component, defect type, etc.), procedure parameters (wave mode, frequency, etc.), and acquisition parameters (sampling rate, averaging rate, bandwidth of receiver, etc.). As a result, the model obtained using FE simulation data is not generic to be used for other geometry or loading configurations, as it may lead to unreliable results. A rational approach of calibrating the baseline model with a specific application structure is to update the model with information measured from that specific structure; therefore, Bayesian updating is proposed to update the baseline model parameters using a few measurement data points from the target structure. 

### 3.3. Bayesian Updating of Baseline Model

Bayesian updating approach combines prior belief (i.e., mathematical model) and new information to obtain a “calibrated” distribution of model parameters [39,40,41,42]. Denote the model parameter as *θ* and its prior distribution as $p_{0}\left(\theta \right)$. Given a likelihood function *L(D|θ)* describing the probability of measurement data *D* and parameter *θ*, the posterior distribution of the parameter, using Bayes’ theorem, reads

(10) $$p\left(\theta \right)\propto p_{0}\left(\theta \right)\cdot L(D|\theta )$$

With the known prior distribution of model parameters and a Gaussian likelihood function, the posterior distribution given a number of *k* measurement from the application structures can be expressed as

(11) $$p\left(a_{1},a_{2},a_{3},a_{4}|D\right)\propto p_{0}\left(a_{1},a_{2},a_{3},a_{4}\right)\cdot \exp\left\{-\frac{1}{2}\overset{k}{\underset{i=1}{\sum }}{\left[\frac{a_{i}-a_{1}-a_{2}x_{i}-a_{3}y_{i}-a_{4}x_{i}y_{i}}{\sigma_{\varepsilon }}\right]}^{2}\right\}$$

where $p_{0}\left(a_{1},a_{2},a_{3}\right)$ is the prior distribution of model parameters estimated using samples drawn from Equation (8), and *σ_ε_* is the standard deviation of the difference between the baseline model prediction of the crack length and the actual measurement of crack length. In Equation (11), a total of *k* measurements of (*a_i_*, *x_i_*, *y_i_*) are used for updating. Without loss of generality, the value of *σ_ε_* can be chosen as the mean value of the samples from the model parameter estimation in previous section. To validate the effectiveness of the Bayesian updating on the baseline model from FE simulation data, two sets of fatigue testing data on actual aluminum plate structures are performed. A few measurement data points are used to perform the Bayesian updating. The crack length prediction results of the updated model are compared with the actual crack lengths. The economic advantage of the overall method is to minimize the cost during baseline model construction. Previous studies use plate-like structures or real components for the training data acquisition. This study uses a numerical FE model for training data; therefore, training data acquisitions on real specimens and components are not needed.

## 4. Experimental Validation

Two groups of in-situ fatigue testing with Lamb wave data acquisition are performed to validate the overall proposed method. Lamb waves are generated and response data are collected using PZT sensors. The pitch-patch configuration is used for Lamb wave data acquisition. Naturally initiated fatigue cracks instead of artificially inserted crack-like defects are developed during the fatigue testing. For each small crack increment, the fatigue testing is paused and the Lamb wave data acquisition is performed. Coupon specimens are used to validate the accuracy of the proposed crack quantification method. Following that, data on realistic lap-joint components with bolt joints are employed to further validate the performance of the proposed method on engineering structures under different loading and damage conditions.

### 4.1. Coupon Application

**Figure 8:**
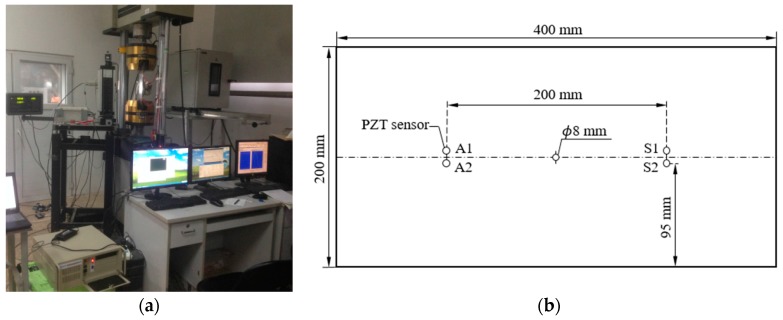
(**a**) Experimental setup of fatigue testing; and (**b**) schematic diagram of specimens and sensors.

The experimental setup for coupon test is shown in Figure 8a. The specimens are made of 2024-T3 aluminum with one hole in the center of the plate. The mechanical properties of the specimen are listed in Table 1. The geometry information of specimen is shown in Figure 8b. The crack size is measured by optical microscopy techniques. The piezoelectric wafers A1 and A2 are used as actuators. The piezoelectric wafers S1 and S2 are used as receivers. The excitation frequency is 160 KHz, which is identical with the setting used in the previous FE model. Before starting the fatigue testing, data from the undamaged plate are acquired and regarded as a reference for comparison purposes. For fatigue testing, a constant loading with a 5 Hz of cycling frequency is used. The peak value of constant loading is 80 MPa and the stress ratio is 0.1. The machine is paused as necessary for Lamb wave data acquisition with the loading stayed at 44 MPa. Test 1 represents the data of Lamb wave propagated from actuator A1 to sensor S1. Test 2 represents the data of Lamb wave propagated from actuator A2 to sensor S2. A total of 23 datasets are acquired for Test 1 and Test 2 during fatigue testing as the crack develops and propagates to different lengths.

**Figure 9:**
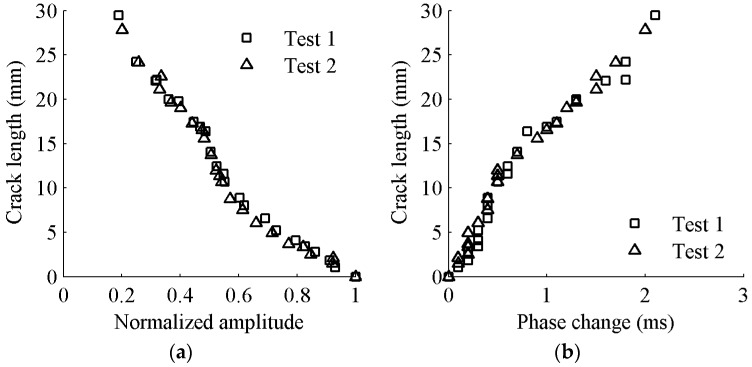
Crack length versus damage sensitive features: (**a**) normalized amplitude; and (**b**) phase change.

The first package of S_0_ mode wave is used to extract the two damage sensitive features (normalized amplitude and phase change). The damage sensitive features at different crack lengths from experiments data are shown in Figure 9. It is observed that the damage sensitive features from experimental data resemble the same monotonic linear relationship as that of FE simulation results. The normalized amplitude decreases and the phase change increases as the crack propagates.

**Figure 10:**
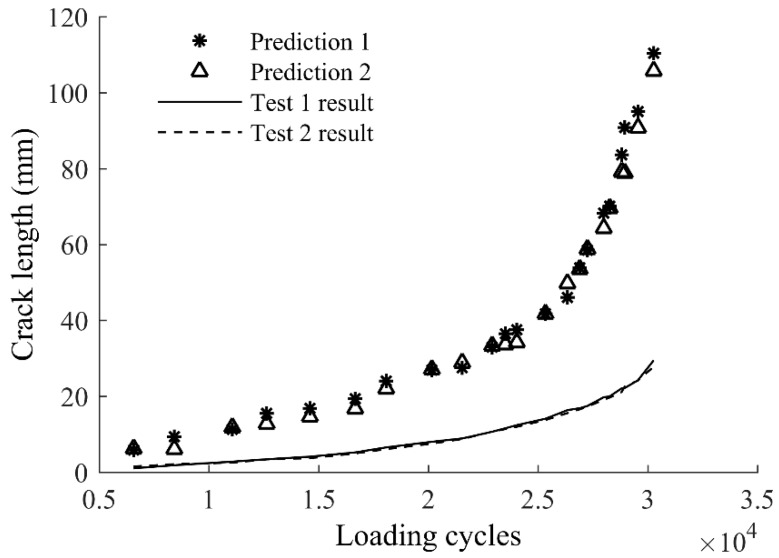
Baseline model predictions of crack growth for Test 1 and Test 2 and experimental results.

To investigate performance of the direct application of the baseline model on coupon components, the damage sensitive features from the acquired Lamb wave data are used in the baseline model, i.e., Equation (9), and the crack size prediction is made. The results are presented in Figure 10, where a large discrepancy between the baseline model prediction and the actual crack growth curve is observed. The large difference indicates that the baseline model from FE modeling only reveals the underlying mechanism of the Lamb wave damage detection and it does not include uncertainties associated with material property, manufacturing, environment, and so on. Direct applications of the baseline model to real structures may lead to unreliable results due to those uncertainties. The baseline model must be further calibrated to a target-specific model for accurate predictions.

**Figure 11:**
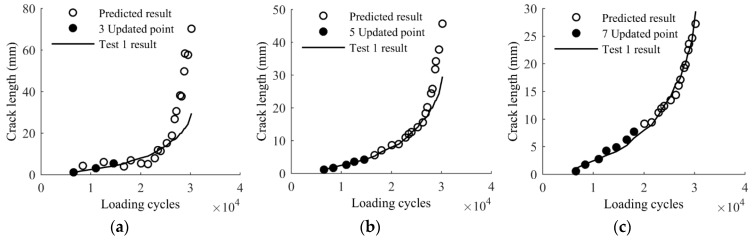
Updated model predictions of crack growth of Test 1: (**a**) updated with three points; (**b**) updated with five points; and (**c**) updated with seven points.

The difference between the prediction from the baseline model and the actual measurements shown in Figure 10 indicates that direct applications of the baseline model are not reliable to obtain accurate results for realistic components; therefore, model calibration is necessary. One rational method for model calibration is using the proposed Bayesian updating with measurement data. To validate the effectiveness of the proposed Bayesian updating for the baseline model, a few measurements of (*a*, *x*, *y*) of Test 1 are used to update the baseline model using Equation (11). The updated model is used to predict the crack length using the extracted (*x*, *y*) from the remaining data of Test 1, and the results are compared with the actual crack size. The crack size predictions using the updated model are further compared with the crack size measurements for the entire Test 2 data. A total number of 1,000,000 samples are generated using MCMC simulations. For each of the samples, a crack size prediction is made using the sample as model parameters and the extracted (*x*, *y*) as model independent variables; therefore, at each of the model updating stage a total number of 1,000,000 model prediction results of crack size can be obtained. For illustration purposes, the median prediction results and the actual crack growth curve are presented in Figure 11. It can be seen that the number of updating points has an influence on the accuracy of predictions. The difference between actual and predicted crack lengths is obvious in Figure 11a since the prior information of the baseline model is dominant and three points for updating is not sufficient to yield accurate results. The prediction gradually converges to the actual crack size with additional updating points, as shown in Figure 11b,c. It can be expected that as more and more structure related specific data points are used for updating, features associated with actual structure will dominate the posterior. As a result the updated model can produce more accurate results compared with the baseline model. It is seen in Figure 11c that the predicted crack lengths and the actual crack lengths are very close, indicating the model updated by seven points is reliable for crack length prediction in this case.

**Figure 12:**
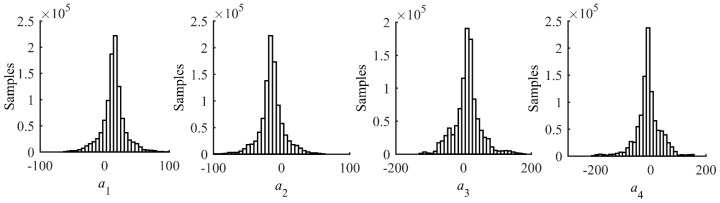
Histograms of the updated model parameters.

Figure 12 shows the posterior distribution of the updated model parameters using seven points. Using the samples, the mean prediction model based on the mean vector of the parameters is obtained as

(12) a=14.4605−14.5193x+8.6573y−6.2484xy

**Figure 13:**
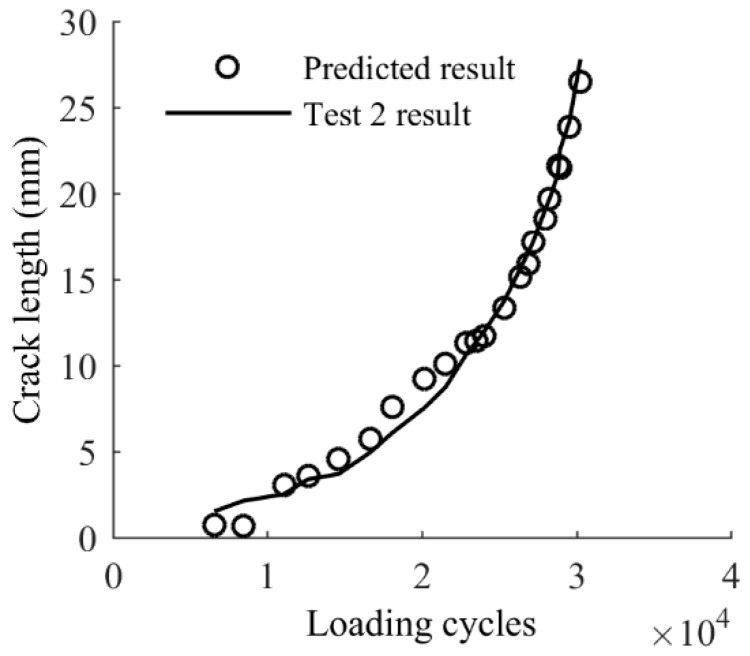
Updated model predictions of crack growth of Test 2.

**Figure 14:**
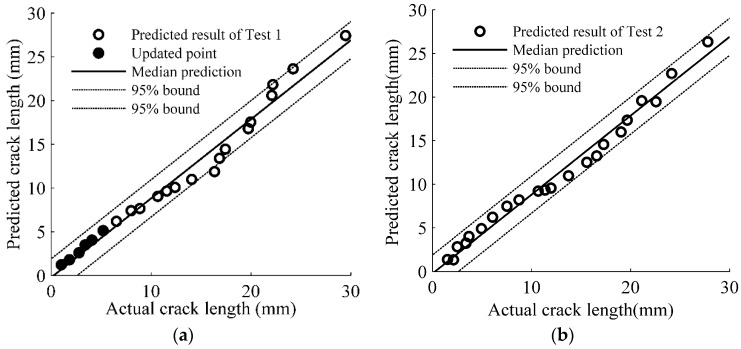
Comparison of updated model predictions and actual measurements: (**a**) Test 1; and (**b**) Test 2.

Equation (12) is employed to predict the crack size for Test 2 and results are shown in Figure 13. A good agreement between the prediction crack size and actual crack size can be observed in Figure 13. Both median prediction and 95% confidence bounds of Test 1 and Test 2 are shown in Figure 14, where most of the data points are within the 95% prediction bounds.

### 4.2. Structural Component Application

To further investigate the effectiveness of the proposed method applied to real-world engineering plate structures under different geometry, material properties, manufacturing, and loading conditions. Data acquired from realistic lap-joint components with naturally developed fatigue crack reported in the previous study are used to validate the accuracy of the proposed method for more complex structures [36].

**Figure 15:**
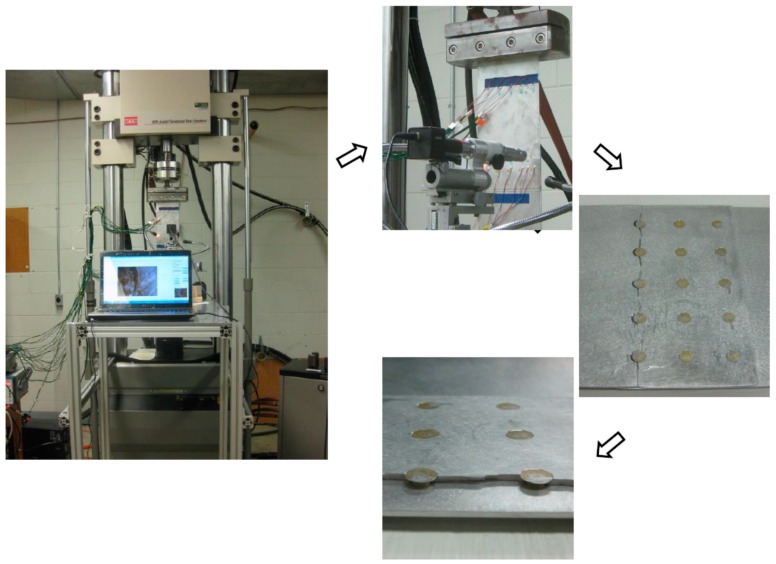
Experimental setup for riveted lap-joint fatigue tests.

**Figure 16:**
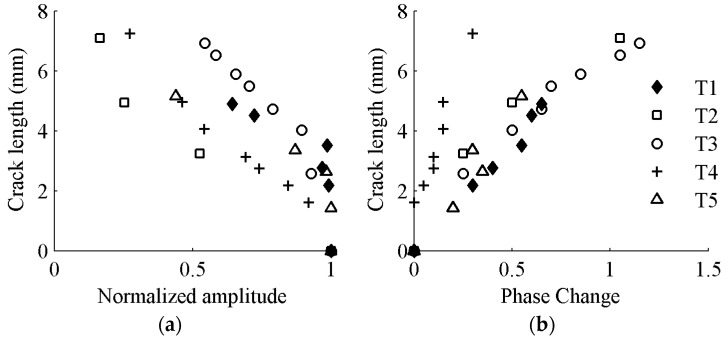
Crack length versus damage sensitive features obtained from in-situ testing of five lap-joint components named T1–T5: (**a**) normalized amplitude; and (**b**) phase change.

The overall experimental setup of the in-situ crack damage quantification of riveted lap-joint components under fatigue loading is shown in Figure 15. It consists of three major parts: (1) the data acquisition system with PZT sensors for Lamb wave signal data generation and acquisition; (2) the fatigue crack optical measurement system, in which a traveling optical microscope with a CCD camera is employed to record the crack propagation during the fatigue testing; and (3) the fatigue cyclic loading system. The lap-joint components are made of 2024-T3 aluminum. PZT sensors are attached on specimens as a pitch-catch configuration. A 3.5-cycle tone burst excitation signal is generated by the actuator, and the response data are received by sensor to interrogate the integrity of the specimen. A band-pass filter is used for de-noising of the raw data. As fatigue loading applied on specimens, the crack is naturally initiated and propagates as the number of loading cycle increases. The details and more information of lap-joint fatigue testing can be referred to Ref. [36]. Damage features are extracted from response signal data acquired during fatigue testing using the same signal processing procedure mentioned in Section 3.1. As shown in Figure 16, the general relationship between the damage sensitive features and the crack sizes are consistent with that obtained in the FE simulation and coupon specimen testing.

**Figure 17:**
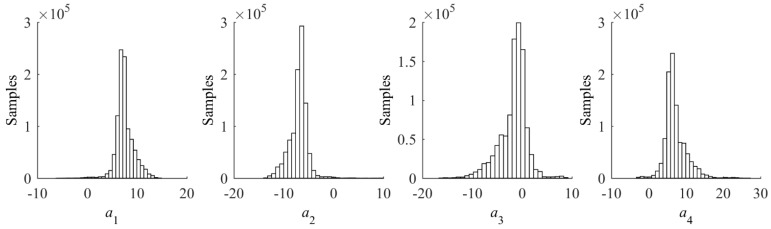
Histograms of the posterior model parameters.

The application example here represents a typical case of using Lamb wave for damage quantification. Because the Lamb wave propagation depends highly on the geometry details of the plate structure of interest, the ideal baseline model calibrated using actual data from the target structure can be time-consuming and expensive. In addition, the local geometry features on plates can vary case by case, and it becomes infeasible to obtain the structure-specific model for every cases. Therefore, the proposed method provides a viable solution for the real applications since it requires numerical experiments to generate the baseline model, which is convenient and easy to perform. As noted before, direct applications of the baseline model based on the FE simulation data may yield unreliable results. The following Bayesian updating using sparse structure specific data can calibrate the model to the structure. The resulting updated model becomes structure specific model and is expected to be accurate and reliable. Data of specimen T1 are used to update the baseline model, and data of the rest specimens are used for model validation. A total number of 1,000,000 MCMC samples are drawn from the posterior distribution, and the resulting histograms of model parameters are presented in Figure 17. The mean prediction model based on the mean values of model parameters is obtained as

(13) a=7.5063−7.0025x−1.7436y+7.1554xy

**Figure 18:**
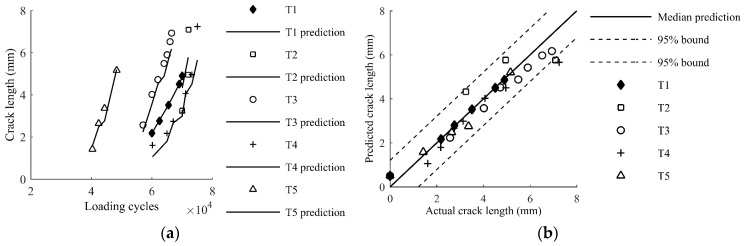
Model predictions and actual measurements of T1–T5: (**a**) crack growth; and (**b**) crack lengths.

Model prediction results for other specimens are presented in Figure 18a, where a satisfactory agreement is observed. Figure 18b presents the detailed prediction results using 95% prediction bounds. It is observed that all data points of T1 are properly overlapped the mean prediction line, because crack features of T1 are used for model updating. Prediction results for other specimens (T2–T5) are close to the mean prediction line and enclosed by the 95% prediction bounds. The comparison demonstrates the overall proposed method is able to quantify crack length for realistic structures using the baseline model from FE data (Figure 2) and sparse measurements from the target structures (Figure 15). To further investigate the performance of the method subject to loading uncertainty and manufacturing uncertainty, two additional testing datasets are used. One dataset is obtained using a different loading spectrum and the other is obtained using a specimen provided by a different manufacture.

#### 4.2.1. Different Loading Spectrum

**Figure 19:**
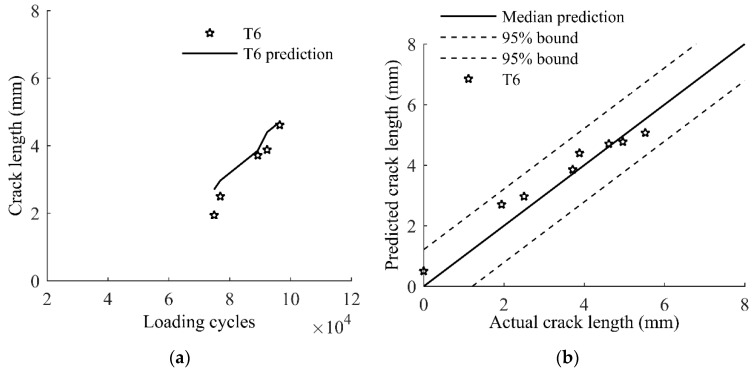
Model predictions and actual measurements of T6: (**a**) crack growth; and (**b**) crack lengths.

The constant fatigue loading is used for specimen T1–T5. A block loading spectrum with an overload is applied to specimen T6. The updated model, Equation (13), is used to predict the crack size for specimen T6. Figure 19 compares the model predictions of the crack size and the actual measurement of crack size. The prediction can still capture the crack size reliably as shown in Figure 19a, and the 95% prediction bounds encloses all the measurement data as shown in Figure 19b.

#### 4.2.2. Specimen from a Different Manufacturer

**Figure 20:**
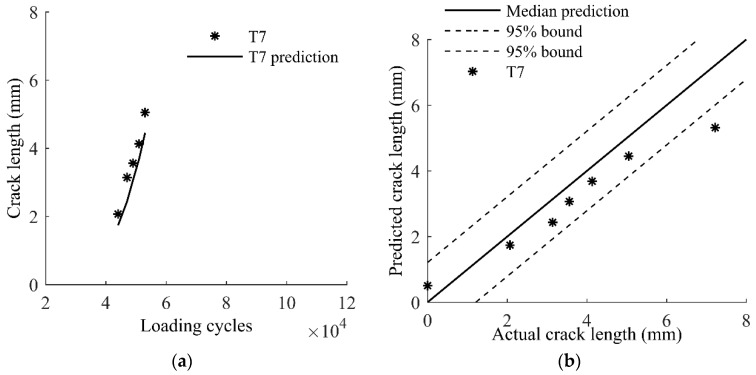
Model predictions and actual measurements of T7: (**a**) crack growth; and (**b**) crack lengths.

Specimens T1–T6 are made by the same manufacturer. An additional specimen T7 from a different manufacture is used to investigate the influence of manufacturing uncertainty to the proposed method. The same constant loading for T1–T5 is used for T6. Results are shown in Figure 20, where a good agreement between the prediction and actual measurements is observed.

## 5. Conclusions

This paper presents an integrated method for crack quantification in plate structures using Lamb wave testing. The method consists of a baseline model built from FE simulation data and Bayesian updating with limited data acquired from the target structure. The baseline model combines two proposed damage sensitive features to formulate a response surface model. The two sensitive features, namely the normalized amplitude and the phase change, extracted from the first received S_0_ wave package, are chosen based on the physical mechanisms of the interaction between Lamb wave propagation and the crack damage. Since the nature of Lamb wave propagation is highly dependent on the local geometry features of the target structure, using FE simulation data to build the baseline quantification model allows for efficient and economic characterization of Lamb wave propagation in the structures. It can flexibly be adapted to new structures with minimal efforts and avoid time-consuming and expensive data acquisition from the actual structure. Due to the difference between FE simulation data and realistic testing data, the baseline model is updated using a few data points from the actual structure. Bayesian updating with MCMC simulations are used. The updated model is expected to be adapted to the specific target structure and provide reliable prediction results. The overall proposed method is rigorously validated using both coupon specimens and realistic lap-joint components. The testing is performed in an in-situ fatigue testing environment to represent the real-world application scenarios. Uncertainties from numerical simulation, loading condition, manufacturing, model parameter, materials are considered during the validation. Based on the current study, conclusions are drawn.

(1) The baseline model using FE simulation data can reliably identify the relationship between the damage sensitive features and the crack length. It provides an economic and efficient way to generate data for the development of damage quantification models.
(2) Direct use of baseline model for realistic application may lead to unreliable results, and the baseline model must be further calibrated to a target-specific model. Bayesian updating provides a rational way to update the initial baseline model with Lamb wave data acquired from the specific target structure. As more data points are used for updating, the model can be calibrated to the target structure and yield accurate prediction results.
(3) The overall method is validated using testing data from coupon specimens and lap-joint components. Due to Bayesian updating, uncertainties from numerical simulation, loading condition, manufacturing, and materials can be included. Validation results indicates the proposed method provide a systematic solution to crack size quantification in plate-like structures.

## Figures and Tables

**Table 1:** Mechanical properties of 2024-T3 aluminum alloy plates.

Material	E (GPa)	G (GPa)	v	ρ (kg/m^3^)
Al2024-T3	72	27	0.33	2780
